# Synthesis and Complex Dielectric Properties of Ba_0.4_Sr_0.6_SnO_3_ Ceramics with Thorn-like Microstructure

**DOI:** 10.3390/ma17246286

**Published:** 2024-12-23

**Authors:** Wei Li, Xiaoyu Wu, Ziheng Huang, Depeng Wang, Weitian Wang

**Affiliations:** School of Physics and Electronic Information, Yantai University, Yantai 264005, China; liwei2024@s.ytu.edu.cn (W.L.); xiaoyuer@s.ytu.edu.cn (X.W.); 1098929197@s.ytu.edu.cn (Z.H.); depengwang@s.ytu.edu.cn (D.W.)

**Keywords:** thorn-like structure, ceramics, dielectric properties, impedance analysis

## Abstract

In this study, we synthesized perovskite Ba_0.4_Sr_0.6_SnO_3_ ceramics with a unique thorn-like microstructure using the solid-state reaction method. The structural and complex dielectric properties were investigated in detail. X-ray diffraction was employed to characterize the phase purity, while X-ray photoelectron spectroscopy was used to analyze the chemical state of the components. The frequency and temperature dependence of the dielectric properties indicates that both the dielectric constant and loss are influenced by A-site ion doping as well as the presence of the thorn-like microstructure. The observed dielectric behavior can be explained by the interfacial polarization and dielectric relaxation processes, which arise from the existing Sn^4+^-Sn^2+^ pairs, oxygen vacancies, and defects with activation energies of 0.38 eV, 0.73 eV, and 0.54 eV, respectively. The resistances of grain boundaries, grains, and the thorn-like structure were revealed by the impedance spectra. These findings provide valuable insights into understanding structure–property relationships in perovskite stannate ceramics.

## 1. Introduction

Perovskite oxides with the general formula ABO_3_ are a class of materials that have gained significant attention in recent years due to their favorable optoelectronic, ferroelectric, and dielectric properties. One of the more appealing features of these materials lies in the fact that they can be easily modified by doping or alloying with other elements, which allows for the tuning of their properties for specific applications. It is widely known that the dielectric properties of perovskite oxides are strongly influenced by their composition. For example, materials with higher concentrations of metal cations tend to have higher permittivity values, while those with lower concentrations of metal cations have lower permittivity values [1,2,3].

In the family of ABO_3_, strontium stannate (SrSnO_3_, SSO) is considered to be a promising material for optoelectronic devices due to its excellent thermal stability and dielectric properties, and the optical, electrical, and semiconductor properties of SSO have been widely studied [4,5,6,7]. It is believed that A-site or B-site doping, defects, and the tilting of the oxygen octahedra are the primary factors affecting the electrical and dielectric performances. By adjusting experimental conditions, such as sintering temperature, cooling rate, or cationic dopings, the crystal structure of SSO can be optimized to enhance the dielectric constant and reduce dielectric loss, which broadens its application potential in electronic components, especially high-performance capacitors [8].

Recently, Singh et al. [9] have reported that electrons and carriers are conducted through oxygen vacancies generated during the sintering process of Ti-doped SSO. Kumar et al. [10] have discovered that Sn^4+^ defects produced by Ti doping can enhance the polarization effect while increasing the dielectric constant. Ouni et al. [11] have demonstrated that Er doping induces transitions due to the tilting of oxygen octahedra, which consistently results in displacement within the perovskite structure, leading to an increase in the dielectric constant and a reduction in dielectric loss. These findings motivate us to explore the effect of A-site doping on the dielectric properties of SSO ceramics and to understand the physical origins.

In this study, Ba-doped SrSnO_3_ (Ba_0.4_Sr_0.6_SnO_3_, BSSO) ceramics were prepared via solid-state reaction method. The phase structure and chemical state of the component elements were investigated using X-ray diffraction (XRD) and X-ray photoelectron spectroscopy (XPS), respectively. The dielectric properties and impedance spectra of the samples were examined as functions of frequency and temperature.

## 2. Experimental Procedure

By employing high-purity chemical reagents BaCO_3_ (≥99.9%), SrCO_3_ (≥99.9%), and SnO_2_ (≥99.5%), the BSSO ceramics were synthesized using the solid-state reaction method. The raw chemical materials were meticulously weighed according to the stoichiometric ratio and thoroughly mixed. The mixed powders were finely ground and subjected to a solid-state calcination process at 1250 °C in a muffle furnace for a duration of 8 h. The chemical equation can be expressed as

(1) $$0.4BaCO_{3}+0.6SrCO_{3}+SnO_{2}\overset{1250\;{}^{\circ}C}{\to }Ba_{0.4}Sr_{0.6}SnO_{3}+CO_{2}$$

Subsequently, the derived materials were reground in an agate pestle and mortar for a period of 1 h, and then pressed into cylindrical pellets of uniform thickness using a pellet press. This process resulted in the formation of 10 mm diameter pellets with a thickness of 3 mm. These pellets were sintered at 1400 °C for 6 h to form ceramic samples. Finally, silver electrodes were uniformly applied to both sides of the pellets for the test. After undergoing thorough drying, the samples were subjected to meticulous characterization and precise dielectric measurements.

The crystal structure of the prepared samples was Investigated using X-ray diffraction (XRD) with Cu Kα radiation (λ = 1.5406 Å) over a 2θ range of 10~80°, employing an X’Pert3 Powder diffractometer (Panalytical, Almelo, The Netherlands). The structural analysis of the XRD data were performed using the General Structure Analysis System (GSAS) and VESTA (JP-Mineral, Ibaraki, Japan) software (2003). X-ray photoelectron spectroscopy (XPS) analysis was performed utilizing a monochromatic Al Kα X-ray source from the ESCALAB 250Xi, a device manufactured by Thermo Electron Corporation (Waltham, MA, USA) in the USA. The morphology of the sintered ceramics was studied by a field emission scanning electron microscope (SEM, Model regulus 8100, Hitachi Co., Tokyo, Japan). To investigate the dielectric properties, a HIOKI 3532-50 LCR HiTester (Hioki Co., Nagano, Japan) and an HP4194A analyzer (HP Development Company, Palo Alto, CA, USA) were utilized for measurements over a frequency range of 100 Hz to 1 MHz.

## 3. Results and Discussions

### 3.1. XRD Analysis

**Figure 1:**
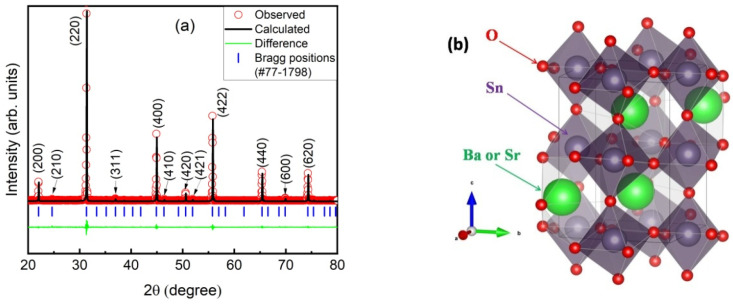
(**a**) Rietveld refinement of XRD pattern of BSSO ceramics. (**b**) Schematic image of crystal structure.

The Rietveld refinement of XRD data are shown in Figure 1a. All the diffraction peaks can be indexed with the standard reference pattern of PDF#77-1798, and the refinement results confirm that the BSSO ceramics crystallize in orthorhombic phase and Pbnm space group with a = 5.752 Å, b = 5.752 Å, and c = 8.078 Å. The obtained unit cell volume is 267.2 Å^3^, a little larger than that of undoped SSO (262.6 Å^3^). This is mainly because the radius of Ba^2+^ is approximately 135 pm, whereas that of Sr^2+^ is approximately 118 pm. Doping with larger ions accounts for the expansion of cell dimensions.

The sketch map of the unit cell structure Is displayed in Figure 1b. The Sn cation (purple) is located at the body-centered cubic (bcc) positions, while Sr or Ba (green) are located at the corner positions and O (red) at the face-centered cubic (fcc) positions. It is believed that A-site doping can modify the crystal structure and charge distribution, leading to enhanced dielectric constants essential for high-performance capacitors and energy storage devices [12].

### 3.2. SEM Images

**Figure 2:**
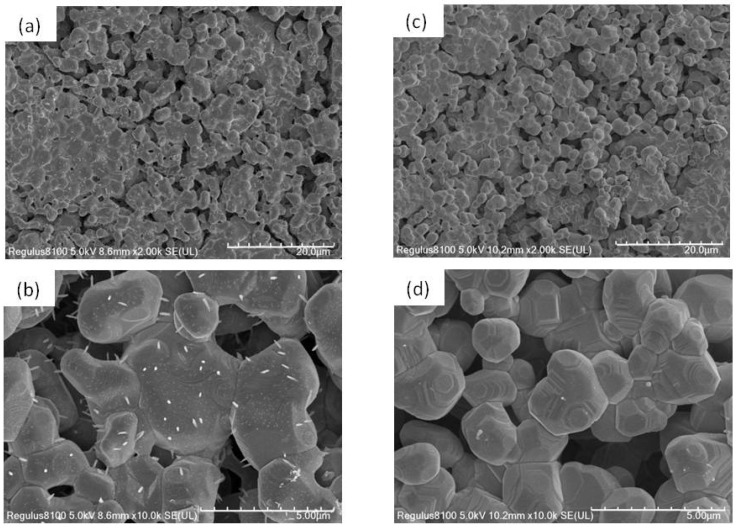
SEM images of (**a**,**b**) BSSO samples, and (**c**,**d**) undoped SrSnO_3_ ceramics.

The SEM images of the prepare BSSO samples are presented in Figure 2a,b. The BSSO samples show a relatively dense microstructure with intergranular porosity. The bulk densities of the samples were measured by the Archimedes method, and the densification of the samples was determined to be about 80~90%. The grain size is about 6–8 μm with narrow size distribution. A careful examination of these micrographs reveals that thorn-like microstructures uniformly distribute on the crystalline grains. SEM images at high magnification (Figure 2b) clearly show the thorn-like morphology with a length of ~1–2 μm. For comparison, SEM images of undoped SSO ceramics prepared under the same experimental conditions are shown in Figure 2c,d. Uniformly distributed grains and grain boundaries can be seen clearly. No noticeable thorn-like microstructures can be identified. Although there are reports about the thorn-like structure grown on the transition metal oxide nanofibers [13,14,15], no similar results have been found in perovskite stannates. Since several review works have reported that group II elements on A-site of perovskite oxides play an important role in the structural and surface evolution activities for their catalytic results [16,17], the doping modification in our experiment can be considered as a catalyst for the formation of the observed thorn-like microstructures. Moreover, anisotropic growth is often favored because it results in a lower overall surface energy. Crystals tend to grow in directions that minimize exposed surfaces with higher surface energies, leading to elongated, thorn-like morphologies.

**Figure 3:**
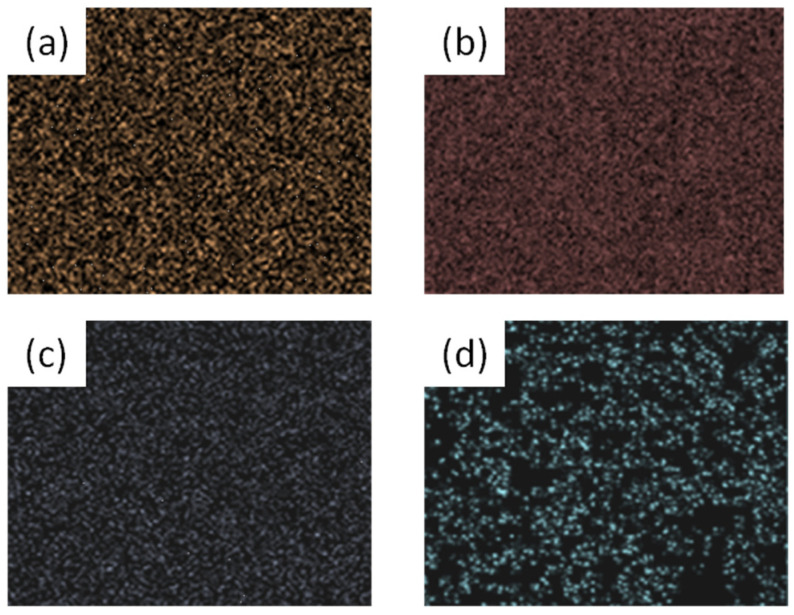
EDS mapping of the BSSO sample: (**a**) Ba, (**b**) Sr, (**c**) Sn, and (**d**) O.

Figure 3 shows the energy-dispersive spectroscopy (EDS) element mapping of BSSO sample. All of the four elements are uniformly distributed without any segregation or aggregation, revealing the chemically homogeneous material. Similar results can be obtained for the undoped SSO ceramics.

### 3.3. XPS Analysis

**Figure 4:**
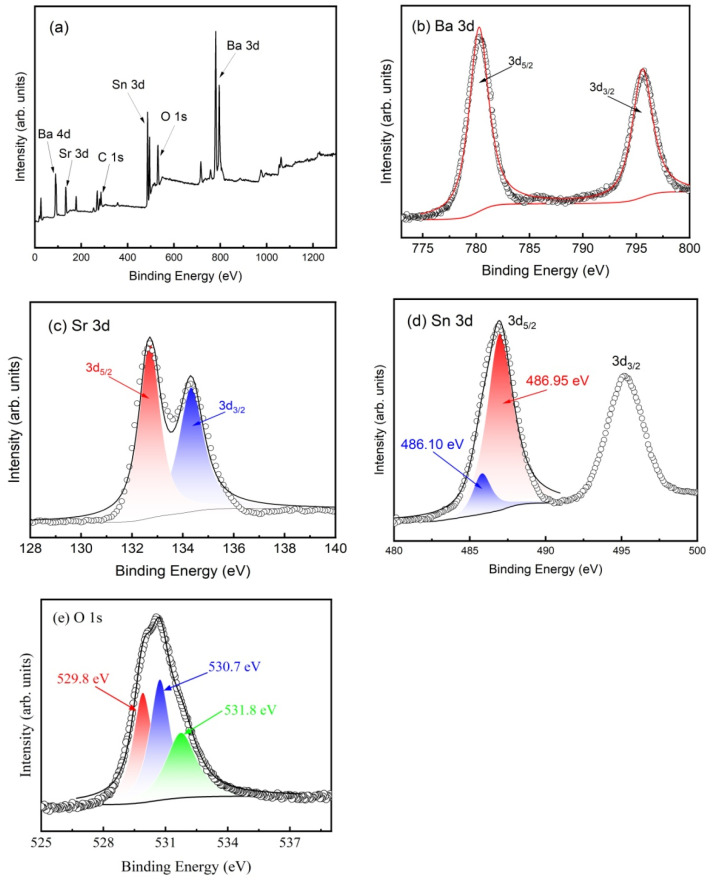
Typical XPS spectra of (**a**) the survey scan, (**b**) Ba 3d, (**c**) Sr 3d, (**d**) Sn 3d, and (**e**) O 1s for the prepared BSSO.

To investigate the chemical state of the component elements, XPS analysis was conducted on the prepared BSSO samples. As shown in Figure 4a, the survey XPS spectrum suggests the presence of Ba, Sr, Sn, O, and C. The binding energy of C 1s at 284.8 eV was used to calibrate the data.

The Ba 3d high-resolution XPS spectrum (Figure 4b) shows characteristic peaks located at 780.3 eV and 795.6 eV, respectively, suggesting that the doping Ba ions are in Ba^2+^ states. The Sr 3d spectrum, shown in Figure 4c, consists of a doublet located at 133.6 eV and 135.4 eV, corresponding to the 3d_5/2_ and 3d_3/2_ states, respectively. The binding energies can be attributed to the Sr^2+^ oxidation state. Figure 4d shows the Sn 3d XPS spectra. The 2d_5/2_ peak is deconvoluted into two peaks located at 486.10 eV and 486.95 eV, attributed to Sn^2+^ and Sn^4+^, respectively. This is in agreement with the reports by Zhong et al. [18], indicating the mixed valence state of tin in the samples. The change in the tin valence state is due to oxygen vacancies formed during the sintering process, which provide additional electrons to the material, reducing Sn^4+^ to Sn^2+^. The existing oxygen vacancies can be confirmed by the O 1s XPS spectra shown in Figure 4e. The asymmetric spectra can be resolved into three Gaussian peaks: lattice oxygen (529.8 eV), oxygen defects (530.7 eV), and surface-adsorbed oxygen (531.8 eV). The results are in line with the literature [19,20,21]. The atomic ratio of Ba:Sr:Sn:O, which can be derived from the XPS data, was calculated to be about 2:3:5:14.7, which is basically consistent with the chemical composition of the BSSO sample.

### 3.4. Dielectric Measurements

**Figure 5:**
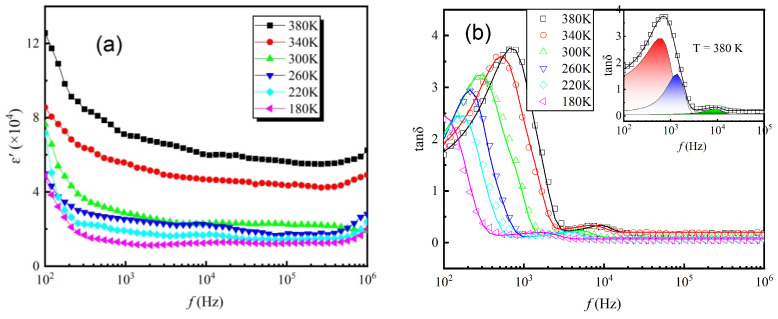
Variations in (**a**) ε′ and (**b**) tanδ as a function of frequency at different temperatures. The solid curves (**b**) are least-squares fitted to tanδ data by using three Gaussian peaks. The inset shows the typical result at *T* = 380 K, and the green, blue, and red peaks represent the high-, medium-, and low-frequency relaxations, respectively.

The frequency dependence of the real part of the dielectric constant ($\varepsilon^{\prime }$) and tangent loss (tanδ) are illustrated in Figure 5. As shown in Figure 5a, the dielectric constant at lower frequencies is large due to the dipolar polarization in the dielectric material. As frequency increases, the dielectric constant tends to reduce because electron movement may not synchronize with the fluctuations of the external electric field, potentially resulting in saturation and minor alterations [22,23]. At fixed frequency, the value of $\varepsilon^{\prime }$ increases with increasing temperature, as a result of the increased thermal energies. The dielectric constant increases from a value of ~1.4 × 10^4^ at about 180 K (1000 Hz) to ~7.3 × 10^4^ at about 380 K (1000 Hz). This phenomenon is mainly caused by the polarization of the associated mobile charge carriers in the studied sample, consistent with the Maxwell–Wagner model [24]. Electron hopping between the Sn^2+^ ions and Sn^4+^ ions is favorable for dielectric behavior. Similar results have been reported for ferrite oxides in Ref. [25]. Moreover, it has been reported that A-site Ba^2+^ doping causes enhanced dielectric constants by inducing defect dipole in the perovskite structures [26], which accounts for the observed large dielectric constant.

The variations of tanδ in Figure 5b show peaks in the frequency range of 10^2^–10^5^ Hz, indicating the presence of relaxation processes, corresponding to polaron relaxation caused by the exchange of electrons in Sn^2+^-Sn^4+^ ions pairs and oxygen vacancies. When the applied frequency is above 10^5^ Hz, the value of tanδ is almost frequency and temperature independent. After fitting the data, the variations of tanδ can be decomposed into three peaks, corresponding to the relaxations at high-, medium-, and low-frequencies. Typical result for *T* = 380 K is shown in the inset of Figure 5b. As the temperature increases, the respective peak positions shift to higher frequencies. For a relaxation related to thermally activated process, the activation energy *E*_a_ can be calculated by using the Arrhenius law:

(2) $$f=f_{0}\exp\left(\frac{-E_{a}}{k_{B}T}\right),$$

where *f*_0_ stands for a pre-exponential factor, *k_B_* is the Boltzmann parameter, and *T* represents the peak temperature.

**Figure 6:**
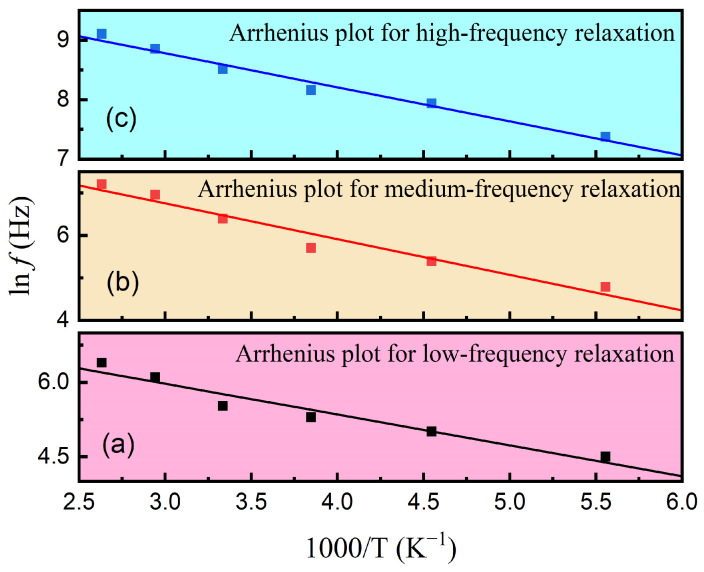
Arrhenius plots for (**a**) the low-frequency relaxation peak, (**b**) the medium-frequency relaxation peak, and (**c**) the high-frequency relaxation peak.

The Arrhenius plots for the three relaxations are shown in Figure 6a–c. The presence of strong linear correlations between 1/*T* and ln*f* indicates the activation energies (*E*_a_) for the high-, medium-, and low-frequency relaxations are 0.38 eV, 0.73 eV, and 0.54 eV, respectively. As reported by Wan and Dang et al. [27,28], 0.3–0.4 eV in the frequency range of 5 × 10^3^~5 × 10^4^ Hz is a typical activation energy for the polaron relaxation caused by the exchange of electrons between multivalent transition metal ions. The obtained 0.38 eV can be attributed to the activated hopping of small polarons between Sn^2+^ and Sn^4+^ ions. The oxide vacancy is a major contributor to the 0.73 eV activation energy [29]. As for the calculated 0.54 eV in the low-frequency region of 10^2^~10^3^ Hz, this activation energy may be related to the relaxations of defects and local effects within the cracks.

**Figure 7:**
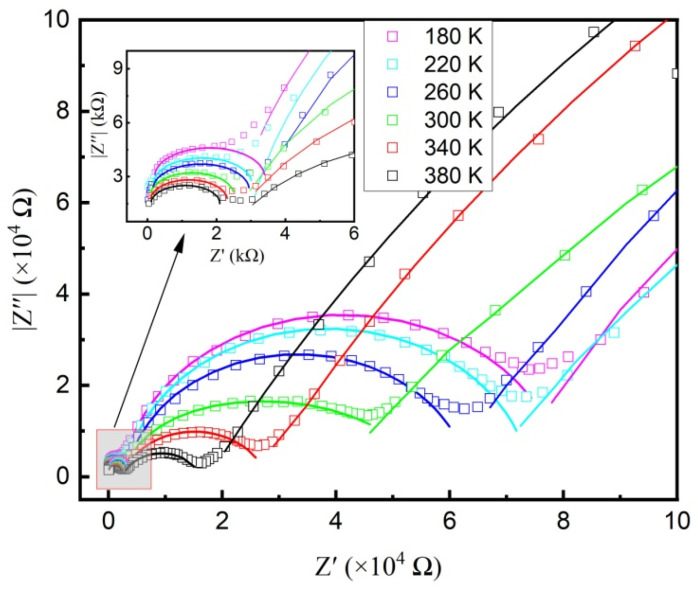
Complex impedance plots for the prepared BSSO ceramics at different temperatures. The solid lines are the best-fitting results.

To separate the dielectric responses from different electroactive regions in the BSSO sample, complex impedance analysis was performed in the temperature range of 180–380 K. The obtained $Z^{\prime }$ vs. $Z^{\prime \prime }$ spectra are presented in Figure 7. Generally, due to the shorter relaxation time of grains compared with that of grain boundaries, the impedance response of grains is located in the high-frequency range, while that of grain boundary is located in the low-frequency range [30]. As for the grain-related microstructures, the responding frequency is higher than that of grains.

**Figure 8:**
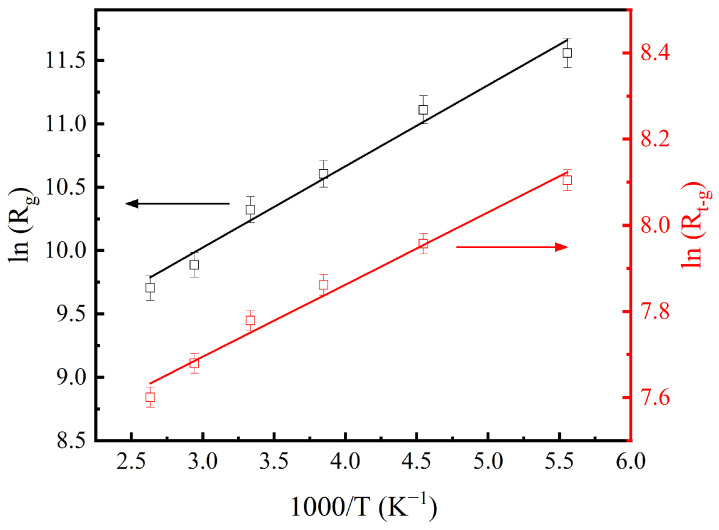
Plots of grain resistance ln(*R*_g_) and the thorn-like structure resistance ln(*R*_t-g_) against 1000/*T*. The solid lines are fitted linearly to the data.

As shown in Figure 7, semicircles with large radii can be observed in the low-frequency region, which can be attributed to the grain boundary response. The presence of intergranular porosity, defects, and oxygen vacancies at the grain boundaries restricts the movement of charge carriers, resulting in the grain boundary impedance being significantly higher than that of grains [31]. The interfaces between grain and grain boundary can act as regions of enhanced polarization due to the interfacial polarization [32]. The determined grain boundary resistance (*R*_gb_) is about 0.2~0.4 MΩ. With the frequency increasing, the radius of the observed semicircles increases with decreasing temperature, which suggests the dependence of the grain resistance on temperature, indicating the semiconducting behavior of grains. As the operating frequency further increases, small semicircles can be found in the high-frequency region, as shown in the inset of Figure 7. The temperature effect on the radius of these semicircles is similar to the results of grains. It is believed that these impedance responses are from the thorn-like microstructures grown on grains. The dependence of the calculated resistance of grains (*R*_g_) and the thorn-like structure (*R*_t-g_) on temperature is shown in Figure 8. As is known, when the mobility of charge carriers is temperature-activated, the resistance *R* depends on the temperature *T*, according to the following equation [33]:

(3) $$R=R_{0}\exp\left(\frac{B}{T}\right),$$

where *R*_0_ and *B* are material-related constants. The linear relationship of ln(*R*_g_)~1/*T* and ln(*R*_t-g_)~1/*T* provides evidence of negative temperature coefficient of resistance, which implies that the prepared BSSO is a stable semiconductor material.

## 4. Conclusions

In conclusion, perovskite Ba_0.4_Sr_0.6_SnO_3_ ceramics with a unique thorn-like microstructure were synthesized using the solid-state reaction method, and the chemical composition, dielectric, and impedance properties were investigated. Dielectric measurements revealed the temperature and frequency dependence of dielectric constant and loss. The relaxations at high-, medium-, and low-frequencies were considered to have originated from the Sn^2+^-Sn^4+^ pairs, oxide vacancy, and defects with activation energies of 0.38 eV, 0.73 eV, and 0.54 eV, respectively. Impedance spectroscopy demonstrated the temperature dependence of grain boundaries, grains, and thorn-like structure responses. The determined grain boundary resistance (*R*_gb_) is about 0.2~0.4 MΩ, while the relationship between the temperature and the resistance of grains and thorn-like structure indicates the semiconducting behavior of the fabricated material. The obtained results suggest that Ba_0.4_Sr_0.6_SnO_3_ ceramics with thorn-like microstructure exhibit excellent dielectric properties due to their unique structure and composition. These properties make them promising candidates for applications in electronic and optoelectronic devices.

## Data Availability

The original contributions presented in the study are included in the article, further inquiries can be directed to the corresponding author.

## References

[1] Ullah K., Khan S.A., Zaman A., Sarker M.R., Ali A., Tirth V., Alsuhaibani A.M., Algahtani A., Al-Mughanam T., Refat M.S. (2023). Impact of cobalt doping on the structural, optical, and dielectric properties of MgAl_2_O_4_ spinel material. ACS Omega.

[2] Kanaun S.K. (2003). Dielectric properties of matrix composite materials with high volume concentrations of inclusions (effective field approach). Int. J. Eng. Sci..

[3] Huang L., Cheng L., Pan S., He Y., Tian C., Yu J., Zhou H. (2020). Effects of Sr doping on the structure, magnetic properties and microwave absorption properties of LaFeO_3_ nanoparticles. Ceram. Int..

[4] Qin X., Li Y., Wu D. (2015). A novel NIR long phosphorescent phosphor: SrSnO_3_: Bi^2+^. RSC Adv..

[5] Aydi A., Khemakhem H., Simon A., Michau D., Mühll R. (2009). Study of ceramic materials in the SrSnO_3_-NaNbO_3_ system by X-ray diffraction, dielectric and Raman spectroscopy. J. Alloys Compd..

[6] Baba E., Kan D., Yamada Y. (2015). Optical and transport properties of transparent conducting La-doped SrSnO_3_ thin films. J. Phys. D Appl. Phys..

[7] Howard C.J., Knight K.S., Kennedy B.J. (2000). The structural phase transitions in strontium zirconate revisited. J. Phys.-Condens. Matter.

[8] Kumar Y., Kumar R., Choudhary R.J. (2020). Reduction in the tilting of oxygen octahedron and its effect on bandgap with La doping in SrSnO_3_. Ceram. Int..

[9] Singh S., Singh P., Parkash O., Kumar D. (2007). Synthesis, microstructure and electrical properties of Ti doped SrSnO_3_. Adv. Appl. Ceram..

[10] Kumar A., Singh M.K., Sharma M., Kumar U. (2022). Structural, dielectric and electrical properties of homovalent doped SrSn_1−x_Ti_x_O_3_ (0 ≤ x ≤ 0.08) system. Indian J. Pure Appl. Phys..

[11] Ouni S., Nouri S., Khemakhem H., Hassen R.B. (2014). Phase transitions, dielectric properties, and vibrational study of stannates perovskites Sr_1−x_Er_x_SnO_3−δ_. Mater. Res. Bull..

[12] Dhahri R., Tayari F., Albargi H.B., Elkenany E.B., Al-Syadi A.M., Sharma N., Lal M., Nassar K.I. (2024). Comprehensive analysis of structural, dielectric, and electrical properties of sol-gel synthesized Ba-doped bismuth ferric titanate perovskite nanoparticles. J. Mater. Sci. Mater. Electron..

[13] Liu Z., Zhao K., Xing G., Zheng W., Tang Y. (2020). One-step synthesis of unique thorn-like BaTiO_3_-TiO_2_ composite nanofibers to enhance piezo-photocatalysis performance. Ceram. Int..

[14] Wu X., Cao L., Song J., Si Y., Yu J., Ding B. (2020). Thorn-like flexible Ag_2_C_2_O_4_/TiO_2_ nanofibers as hierarchical heterojunction photocatalysts for efficient visible-light-driven bacteria-killing. J. Colloid Interface Sci..

[15] Arthi G., Selvam R., Muthamizhchelvan C., Hayakawa Y., Ramaraj S.G. (2023). Thorn-like morphology of TiO_2_ hierarchical structures for efficient dye-sensitized solar cell application. Mater. Lett..

[16] Antipin D., Risch M. (2020). Trends of epitaxial perovskite oxide films catalyzing the oxygen evolution reaction in alkaline media. J. Phys. Energy.

[17] Zhao J.W., Li Y., Luan D., Lou X.W. (2024). Structural evolution and catalytic mechanisms of perovskite oxides in electrocatalysis. Sci. Adv..

[18] Zhong F., Zhuang H., Gu Q. (2016). Structural evolution of alkaline earth metal stannates MSnO_3_ (M = Ca, Sr, and Ba) photocatalysts for hydrogen production. Rsc. Adv..

[19] Ou G., Xu Y., Wen B. (2018). Tuning defects in oxides at room temperature by lithium reduction. Nat. Commun..

[20] Jaim H.M., Lee S., Zhang X. (2017). Stability of the oxygen vacancy induced conductivity in BaSnO_3_ thin films on SrTiO_3_. Appl. Phys. Lett..

[21] Tyuliev G., Angelov S. (1988). The nature of excess oxygen in Co_3_O_4+ϵ_. Appl. Surf. Sci..

[22] Zarrin N., Husain S., Sharma S. (2020). Thermally stimulated small polaron promoted conduction mechanism in Fe-doped La_0.7_Sm_0.3_CrO_3_. J. Phys. Chem. Solids.

[23] Abushad M., Khan W., Naseem S., Husain S., Nadeem M., Ansari A. (2019). Influence of Mn doping on microstructure, optical, dielectric and magnetic properties of BiFeO_3_ nanoceramics synthesized via solgel method. Ceram. Int..

[24] Kumar U., Ankur K., Yadav D., Upadhyay S. (2020). Synthesis and characterization of Ruddlesden-Popper system (Ba_1−x_Sr_x_)_2_SnO_4_. Mater. Charact..

[25] Sagdeo A., Gautam K., Sagdeo P.R., Singh M.N., Gupta S.M., Nigam A.K., Rawat R., Sinha A.K., Ghosh H., Ganguli T. (2014). Large dielectric permittivity and possible correlation between magnetic and dielectric properties in bulk BaFeO_3−δ_. Appl. Phys. Lett..

[26] Balaraman A.A., Dutta S. (2022). Inorganic dielectric materials for energy storage applications: A review. J. Phys. D Appl. Phys..

[27] Wan F., Hua X., Guo Q. (2024). Modulation of the structural, magnetic, and dielectric properties of YMnO_3_ by Cu doping. Materials.

[28] Dang N.T., Kozlenko D.P., Tran N., Lee B.W., Phan T.L., Madhogaria R.P., Kalappattil V., Yang D.S., Kichanov S.E., Lukin E.V. (2019). Structural, magnetic and electronic properties of Ti-doped BaFeO_3−δ_ exhibiting colossal dielectric permittivity. J. Alloys Compd..

[29] Saxena P., Mishra A. (2021). Structural and electrical properties of YMnO_3_ manganites: Influence of Cr ion doping. J. Solid State Chem..

[30] Biswal M.R., Nanda J., Mishra N.C., Anwar S., Mishra A. (2014). Dielectric and impedance spectroscopic studies of multiferroic BiFe_1−x_Ni_x_O_3_. Adv. Mater. Lett..

[31] Futazuka T., Ishikawa R., Shibata N., Ikuhara Y. (2022). Grain boundary structural transformation induced by co-segregation of aliovalent dopants. Nat. Commun..

[32] Feng Y., Li W.L., Hou Y.F., Yu Y., Cao W.P., Zhang T.D., Fei W.D. (2015). Enhanced dielectric properties of PVDFHFP/BaTiO_3_-nanowires composites induced by interfacial polarization and wire-shape. J. Mater. Chem. C.

[33] Feteira A. (2009). Negative temperature coefficient resistance (NTCR) ceramic thermistors: An industrial perspective. J. Am. Ceram. Soc..

